# Microbial synthesis of the plant natural product precursor *p*-coumaric acid with *Corynebacterium glutamicum*

**DOI:** 10.1186/s12934-023-02222-y

**Published:** 2023-10-13

**Authors:** Mario Mutz, Dominic Kösters, Benedikt Wynands, Nick Wierckx, Jan Marienhagen

**Affiliations:** 1 Institute of Bio- and Geosciences, IBG-1: Biotechnology, Forschungszentrum Jülich, 52425 Jülich, Germany; 2 Institute of Biotechnology, RWTH Aachen University, Worringer Weg 3, 52074 Aachen, Germany

**Keywords:** *p*-coumaric acid, Phenylpropanoids, Anthranilate, *Corynebacterium glutamicum*, Shikimate pathway, Feedback inhibition, Co-cultivation, Plant polyphenols, Metabolic engineering

## Abstract

**Background:**

Phenylpropanoids such as *p*-coumaric acid represent important precursors for the synthesis of a broad range of plant secondary metabolites including stilbenoids, flavonoids, and lignans, which are of pharmacological interest due to their health-promoting properties. Although extraction from plant material or chemical synthesis is possible, microbial synthesis of *p*-coumaric acid from glucose has the advantage of being less expensive and more resource efficient. In this study, *Corynebacterium glutamicum* was engineered for the production of the plant polyphenol precursor *p*-coumaric acid from glucose.

**Results:**

Heterologous expression of the tyrosine ammonia-lyase encoding gene from *Flavobacterium johnsoniae* enabled the conversion of endogenously provided tyrosine to *p*-coumaric acid. Product consumption was avoided by abolishing essential reactions of the phenylpropanoid degradation pathway. Accumulation of anthranilate as a major byproduct was eliminated by reducing the activity of anthranilate synthase through targeted mutagenesis to avoid tryptophan auxotrophy. Subsequently, the carbon flux into the shikimate pathway was increased, phenylalanine biosynthesis was reduced, and phosphoenolpyruvate availability was improved to boost *p*-coumaric acid accumulation. A maximum titer of 661 mg/L *p*-coumaric acid (4 mM) in defined mineral medium was reached. Finally, the production strain was utilized in co-cultivations with a *C.* *glutamicum* strain previously engineered for the conversion of *p*-coumaric acid into the polyphenol resveratrol. These co-cultivations enabled the synthesis of 31.2 mg/L (0.14 mM) resveratrol from glucose without any *p*-coumaric acid supplementation.

**Conclusions:**

The utilization of a heterologous tyrosine ammonia-lyase in combination with optimization of the shikimate pathway enabled the efficient production of *p*-coumaric acid with *C. glutamicum*. Reducing the carbon flux into the phenylalanine and tryptophan branches was the key to success along with the introduction of feedback-resistant enzyme variants.

**Supplementary Information:**

The online version contains supplementary material available at 10.1186/s12934-023-02222-y.

## Background

Phenylpropanoids such as *p*-coumaric acid (*p*-CA) are precursors of polyphenols such as stilbenes, chalcones, or flavonoids, which gained pharmacological interest due to antioxidative, antiviral, or anticarcinogenic properties [1]. In addition, they fulfill other functions in plant metabolism as ferulic acid and *p*-CA are covalently bound to polysaccharides of higher plant’s cell walls acting as cross-linkers between lignin polymers, hemicellulose, and cellulose [2]. In plants, phenylpropanoids are also part of the response to biotic and abiotic stimuli such as variation of light and are the main mediators of resistance to pathogens [3]. Moreover, phenolic phytochemicals such as *p*-CA are valued monomers for the synthesis of biodegradable liquid crystal polymers [4, 5]. In order to extract esterified *p*-CA from plant material, ester bonds are typically hydrolyzed by alcohol/water mixtures or alkaline hydrolysis [6, 7]. Hydrolysis results in complex extracts of co-extracted sugars, proteins, and polyphenols, which make further purification necessary [8–10]. Hence, alternative extraction methods such as pressurized liquid extraction, microwaves, or extrusion could be applied, but are limited by the available biomass and the economics of extraction and purification steps [11, 12]. Chemical *p*-CA synthesis is based on Knoevenagel-Doebner condensation of malonate and 4-hydroxybenzaldehyde employing a pyridine catalyst [12]. Even though more sustainable methods under milder reaction conditions have already been developed, chemically synthesized *p*-CA is also classified as non-natural by the cosmetic and food industries and thus would only find limited application [13, 14].

**Fig. 1:**
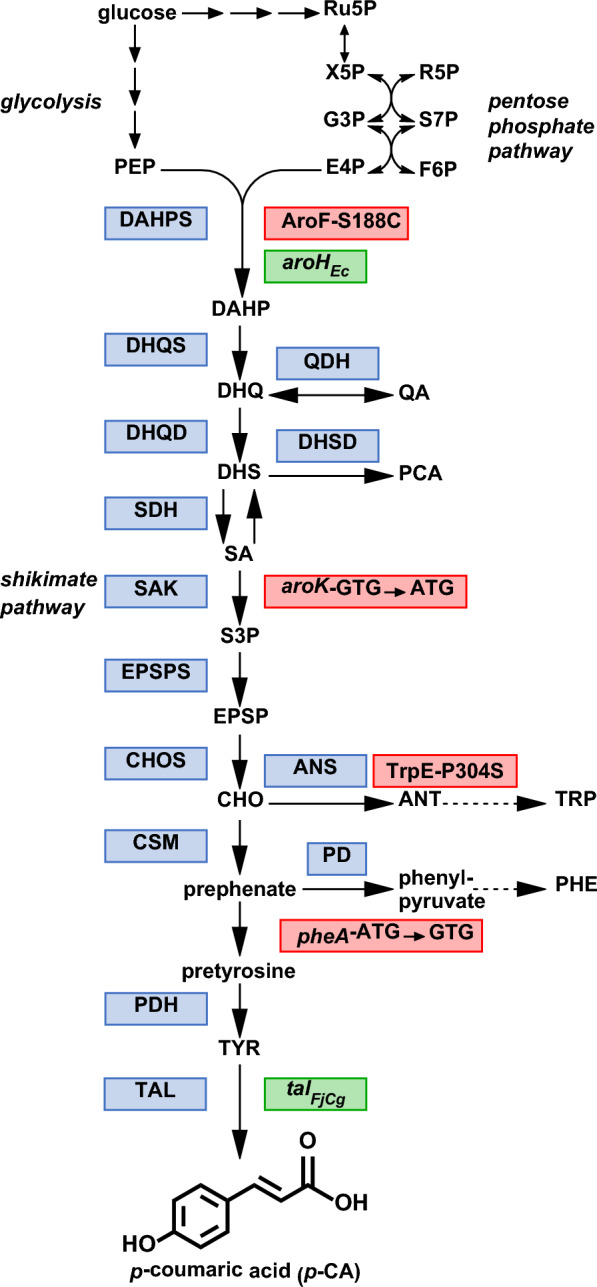
Biosynthesis of *p*-coumaric acid from glucose via the shikimate pathway in *C. glutamicum*. Required enzymes and genes are shown in boxes. Blue Boxes indicate the respective enzymatic activities involved in TYR biosynthesis via the shikimate pathway. Red boxes highlight the modifications of endogenous enzymes or genes, whereas green boxes indicate episomal expression of heterologous genes. Dashed arrows represent two or more catalytic steps. Abbreviations: AroF-S188C, mutated DAHP synthase AroF carrying the amino acid substitution S188C; *aroH*_*Ec*_, DAHP synthase gene from *E. coli* for episomal expression; *ANT* anthranilate, *ANS* anthranilate synthase, *aroK-GTG*→*ATG* gene encoding shikimate kinase with start codon replacement (GTG→ATG), *CHOS* chorismate synthase, *CSM* chorismate mutase, *DAHP* 3-deoxy-arabino-heptulosonate-7-phosphate, *DAHPS* DAHP synthase, *DHQD* 3-dehydroquinate dehydratase, *DHQS* 3-dehydroquinate synthase, *DHSD* 3-dehydroshikimate dehydratase, *E4P* erythrose-4-phosphate, *EPSP* 5-enolpyruvylshikimate-3-phosphate, *EPSPS* EPSP synthase, *F6P* fructose-6-phosphate, *G3P* glyceraldehyde-3-phosphate, *PCA* protocatechuate, *PD* prephenate dehydratase, *PDH* pretyrosine dehydrogenase, *PEP* phosphoenolpyruvate, *pheA-ATG*→*GTG* gene encoding prephenate dehydratase with start codon replacement (ATG→GTG), *QA* quinate, *QDH* quinate dehydrogenase, *Ru5P* ribulose-5-phosphate, *R5P* ribose-5-phosphate, *SA* shikimate, *SAK* shikimate kinase, *SDH* shikimate dehydrogenase, *S7P* sedoheptulose-7-phosphate, *TAL* tyrosine ammonia-lyase, *tal*_*FjCg*_ codon-optimized gene encoding tyrosine ammonia-lyase from *Flavobacterium johnsoniae* for episomal expression, *TrpE-P304S* mutated anthranilate synthase component I (TrpE) carrying the amino acid substitution P304S, *TRP* tryptophan TYR, tyrosine, *X5P* xylulose-5-phosphate

As an alternative, *p*-CA could also be produced in a more economical and environmentally friendly way from cheap carbon sources using microorganisms. In plants and bacteria, the direct *p*-CA precursor tyrosine (TYR) is synthesized via the shikimate (SA) pathway, which provides all aromatic amino acids and other important aromatic compounds [15] (Fig. 1).

The first reaction of this pathway is the condensation of erythrose-4-phosphate (E4P) and phosphoenolpyruvate (PEP) to 3-deoxy-arabino-heptulosonate-7-phosphate (DAHP) catalyzed by DAHP synthase [16]. DAHP is converted to chorismate (CHO) in six enzymatic reactions, which represents a branching point in microbial metabolism. CHO can be converted to anthranilate (ANT) by ANT synthase (ANS), which is the first step of tryptophan (TRP) biosynthesis. Alternatively, CHO can also be converted to prephenate by the activity of chorismate mutase (CSM) representing the precursor of phenylalanine (PHE) and TYR [17, 18]. In plants, the amino group of PHE and TYR is non-oxidatively eliminated by phenylalanine ammonia-lyase (PAL) or tyrosine ammonia-lyase (TAL) giving rise to the phenylpropanoids cinnamate and *p*-CA, whereby cinnamate can be hydroxylated by a cinnamate 4-hydroxylase (C4H) to *p*-CA [19, 20]. As a TAL-activity is mostly absent in non-model organisms, the microbial synthesis of *p*-CA or *p-*CA-derived products from glucose or xylose is typically enabled by heterologous expression of TAL-encoding genes, e.g. in *Escherichia coli*, *Pseudomonas putida*, *P. taiwanensis* or *Saccharomyces cerevisiae* [5, 21–27]. A product titer of 974 mg/L *p-*CA was achieved with *E. coli* upon expression of a codon-optimized *tal* gene and deregulation of TYR biosynthesis [5]. *P. putida* and *S. cerevisiae* strains are described that achieved *p*-CA titers of 1.7 or 12.5 g/L by fed-batch cultivations in bioreactors, demonstrating the great potential of microorganisms for *p*-CA production [23, 28].

Another suitable platform organism for *p*-CA production is the gram-positive soil bacterium *Corynebacterium glutamicum* since corn starch-derived products obtained from *C. glutamicum* cultivations have the GRAS status [29]. Thus, as a workhorse of industrial biotechnology, *C. glutamicum* is utilized for the production of proteinogenic amino acids such as glutamate and lysine at a scale of several million tons per year [30]. In addition, *C.* *glutamicum* strains for the synthesis of a broad variety of *p*-CA-derived plant natural products (PNPs) such as anthocyanins, resveratrol (RES), raspberry ketone or naringenin are available [31–34]*.* PNP production with *C. glutamicum* typically relies on *p*-CA precursor feeding, which is expensive (115–330 $/kg), rendering supplementation of this phenylpropanoid undesirable for any large-scale production of *p*-CA-derived products [12].

In this study, *p*-CA production from glucose was established with *C.* *glutamicum* by pursuing different rational metabolic engineering strategies. The specific product formation was improved by adjusting the inorganic phosphate (P_i_) concentration of the mineral medium. In co-cultivations with a PNP-producing *C.* *glutamicum* variant, the best *p*-CA production strain was used for the microbial production of RES from glucose.

## Results

### Evaluation of p-CA toxicity

Previously, *C.* *glutamicum* DelAro^5^ C7 P_O6_-*iolT1* (*C.* *glutamicum p*-CA1) was constructed for the microbial production of aromatic compounds such as hydroxybenzoic acids [35]. Deletion of 27 genes in five gene clusters involved in the catabolism of aromatic compounds renders this strain suitable for the production of *p*-CA. Moreover, the native promotor of *gltA* encoding citrate synthase (CS) was replaced by the *dapA* promotor variant C7 in this strain in order to reduce CS activity and to increase PEP availability, which represents an important precursor molecule of the SA pathway [36]. Finally, deregulation of the *iolT1*-gene realized by two point mutations in the promoter of this gene proved to be beneficial for increased uptake of glucose via the *myo-*inositol transporter IolT1 (P_O6_-*iolT1*).

**Fig. 2:**
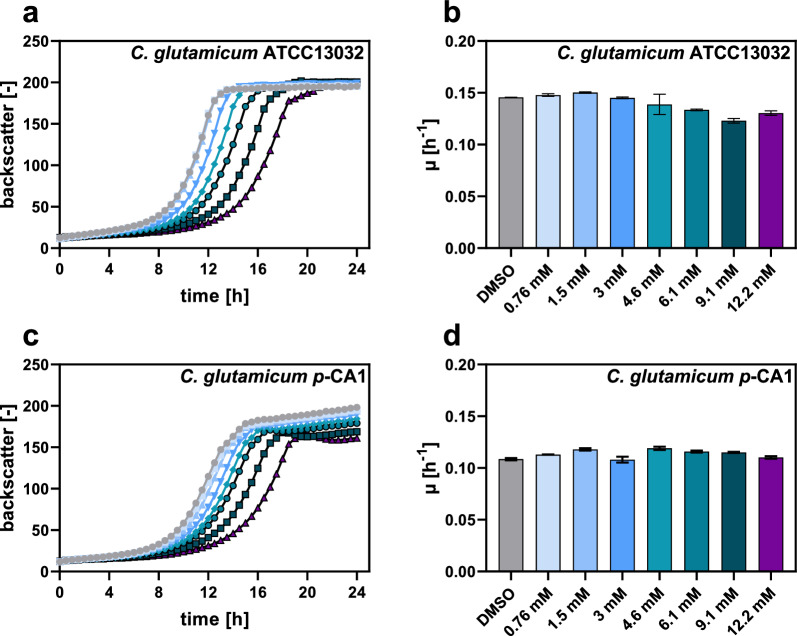
Cytotoxic effects of *p*-CA on the growth of *C. glutamicum* ATCC13032 and *C. glutamicum p*-CA1. **a**, **c** Growth and **b**, **d** determined growth rates of *C. glutamicum* ATCC13032 and *C. glutamicum p*-CA1 in the presence of increasing *p*-CA concentrations are depicted. As a control, 20 µL DMSO was added. The data represent the average and standard deviation of biological triplicates

With the aim to test the suitability of *C.* *glutamicum* for the production of *p*-CA, the toxicity of *p*-CA on microbial growth was evaluated. For this purpose, the *C. glutamicum* ATCC13032 wild type and the production host *C. glutamicum p*-CA1 were cultivated in microtiter plates (MTPs) in the presence of 1.5–12.2 mM *p*-CA (Fig. 2).

In these experiments, growth of the *C.* *glutamicum* wild type was characterized by longer lag-phases with increasing *p*-CA concentrations and the growth rate dropped to 0.11 h^−1^ at *p*-CA concentration exceeding 9.1 mM compared to the control (0.15 h^−1^) without *p*-CA. *C. glutamicum p*-CA1 showed a similar growth behavior, even though the overall growth rate with 0.10 h^−1^ was significantly lower compared to the wild type. Interestingly, the final biomass reached for *C. glutamicum p*-CA1 appeared to be a slightly reduced in the presence of higher *p*-CA concentrations. However, for this strain no reduction of the growth rate could be observed at the *p*-CA concentrations tested, *C. glutamicum p*-CA1 a suitable host system for the production of *p*-CA.

### Establishing *p*-CA production and prevention of product degradation

**Fig. 3:**
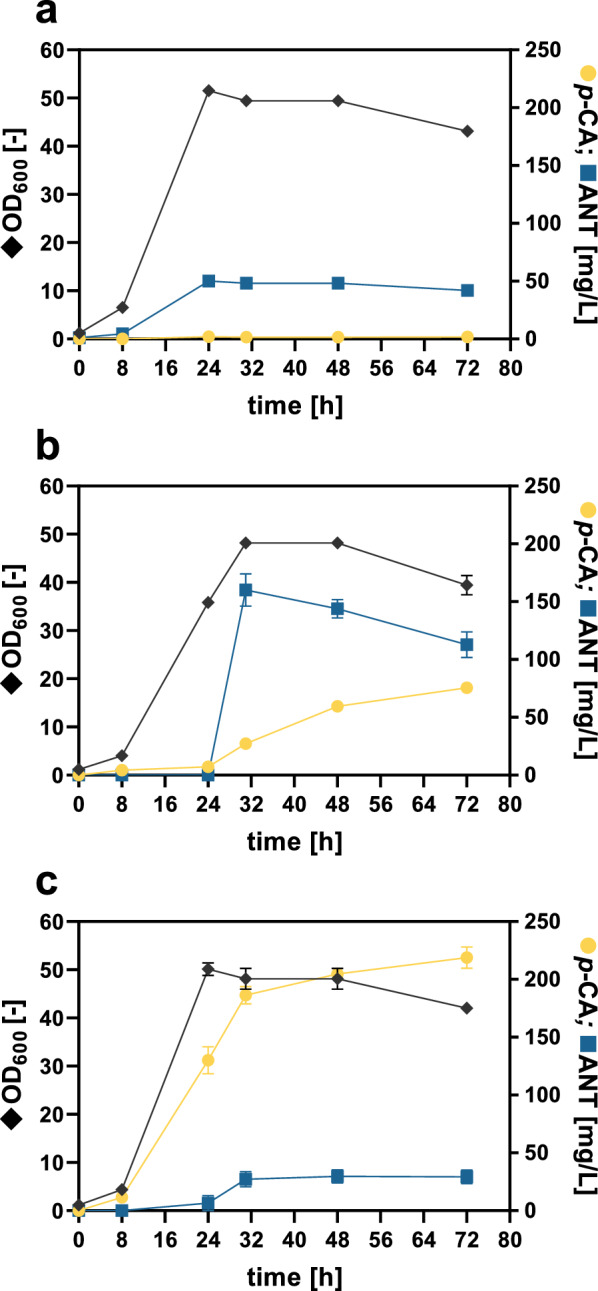
*p*-CA production with *C. glutamicum* and effect of the *phdA*-deletion on *p*-CA synthesis. Growth (OD_600_), *p*-CA titer, and ANT concentration of (**a**) *C. glutamicum p*-CA1 harboring the empty vector pEKEx3, (**b**) *C. glutamicum p*-CA1 pEKEx3-*aroH*_*Ec*_-*tal*_*FjCg*_ and (**c**) *C. glutamicum p*-CA1 ∆*phdA* (*C. glutamicum p*-CA2) pEKEx3-*aroH*_*Ec*_-*tal*_*FjCg*_. The depicted data represent mean values and standard deviation of biological triplicates

The production of *p*-CA was established by episomal heterologous expression of *aroH*_*Ec*_ encoding a DAHP synthase from *E. coli* and the codon-optimized *tal*_*FjCg*_ gene encoding the TAL from *Flavobacterium johnsoniae* [31]*.* For this, *C. glutamicum p*-CA1 harboring the empty vector or the expression plasmid pEKEx3-*aroH*_*Ec*_-*tal*_*FjCg*_ was cultivated in shake flasks with glucose as the sole carbon and energy source (Fig. 3a, b).

A *p*-CA titer of 75 ± 0.3 mg/L (0.46 mM) could be determined after 72 h of cultivation, whereas a control strain carrying only the empty vector showed no *p*-CA production. While after 24 h the control strain reached an OD_600_ of 51.5 ± 0.9, the growth of the *p*-CA-producing strain was slower (35.8 ± 0.3 after 24 h), which increased to 48.2 ± 1.1 after 32 h of cultivation. Surprisingly, accumulation of the TRP pathway intermediate ANT was observed as a major byproduct (160 ± 13.9 mg/L (1.2 mM)), withdrawing the *p*-CA-precursor CHO.

During the development of the *C.* *glutamicum* DelAro^5^ platform strain for the production of stilbenes and other polyphenols, the *phd* gene cluster was deleted, which is required for the degradation of phenylpropanoids in *C. glutamicum* [37]. However, in this process, the genes *phdA* and *phdT* encoding an acyl:CoA ligase (PhdA) and a phenylpropanoid transporter (PhdT), respectively, were not deleted as both enzyme functions could be exploited for plant polyphenol production with *C.* *glutamicum* [31]. PhdA catalyzes the CoA-activation of *p*-CA to the phenylpropanoyl-CoA thioester *p*-coumaroyl-CoA, which mediates feedback-inhibition of the TAL resulting in a decreased conversion of TYR to *p*-CA [38]. In order to avoid CoA-activation of *p*-CA, *phdA* was deleted in *C. glutamicum p*-CA1 (*C. glutamicum p*-CA2). The effect of this deletion on *p*-CA production and growth was evaluated in shake flasks (Fig. 3c). The *phdA*-deficient strain demonstrated faster biomass formation compared to the parental strain and reached the stationary growth phase after 24 h. Production of *p*-CA started already after 8 h of cultivation and a maximum titer of 218 ± 9.3 mg/L (1.3 mM) *p*-CA could be determined so that the product titer was markedly increased compared to the parental strain still carrying *phdA*. This suggests that a fraction of produced *p*-CA is converted to its corresponding thioester in the latter strain. However, *p*-coumaroyl-CoA cannot be detected by available GC-TOF MS methods, probably due to the observed spontaneous hydrolysis of CoA-activated thioesters releasing *p*-CA as free acid during GC-TOF MS analysis [37].

### Modulation of the carbon flux into the shikimate pathway and pathways for aromatic amino acids

Next, attempts were made to direct the carbon flow through the SA pathway towards TYR as a direct precursor of *p*-CA. By converting prephenate to phenylpyruvate and finally to PHE, precursors are withdrawn from the desired *p*-CA synthesis, which limits overall *p*-CA production. In order to reduce the initial translation efficiency of the prephenate dehydratase gene *pheA*, the translational start codon was replaced (ATG→GTG). Elimination of this essential enzyme activity was refrained from, as the resulting PHE auxotrophy would have necessitated supplementation of PHE. *C. glutamicum* possesses two DAHP synthase isozymes. AroG is feedback-inhibited by TRP and AroF by TYR [39, 40]. In a parallel approach, it was attempted to increase the carbon flux into the SA pathway by eliminating TYR-mediated feedback-inhibition of AroF by introducing a point mutation in the genomic *aroF* gene copy resulting in the amino acid substitution S188C (AroF-S188C) [41]. Both modifications, the start codon replacement of *pheA* and introduction of the mutation into AroF were introduced separately and in combination to investigate possible synergistic effects on *p*-CA and byproduct formation (Additional file 1: Fig. S1). The start codon replacement of *pheA* (ATG→GTG) resulted in the formation of 442 ± 50.9 mg/L (2.2 mM) *p*-CA doubling the product titer. The relief of feedback inhibition of AroF and a combination of both modifications allowed for the same product titer (469 ± 10.1 mg/L (2.9 mM) and 470 ± 13 mg/L (2.9 mM), respectively). However, as observed previously, all strains also accumulated ANT as a major byproduct. The highest ANT concentration could be determined in supernatants of the variant strain harboring the AroF-S188C substitution with 637 ± 26.6 mg/L (4.6 mM). The strain *C. glutamicum p*-CA2 GTG-*pheA* AroF-S188C (*C. glutamicum p*-CA3) was used for further strain engineering as it allowed for the highest product titer and the lowest ANT accumulation with 305 ± 56.7 mg/L (2.2 mM).

**Fig. 4:**
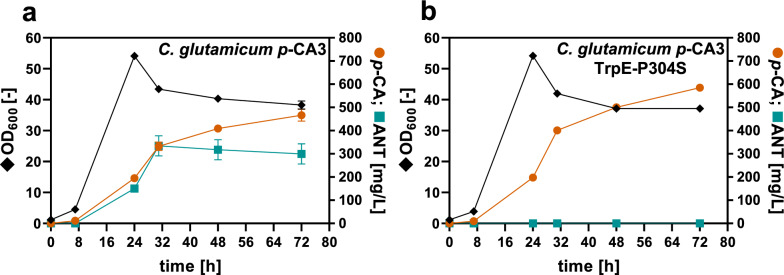
Effect of amino acid substitution P304S in anthranilate synthase component I (TrpE-P304S) on *p*-CA production and anthranilate byproduct formation. Growth (OD_600_) (diamonds), *p*-CA (circles), and ANT titer (squares) of (**a**) *C. glutamicum p*-CA3 and (**b**) *C. glutamicum p*-CA4 with mutated anthranilate synthase component I (TrpE-P304S). All strains harbor the expression plasmid pEKEx3-*aroH*_*Ec*_*-tal*_*FjCg*_ for *p*-CA production. The depicted data represent mean values and standard deviation of biological triplicates

In order to reduce ANT formation and redirect the carbon flux toward *p*-CA, the conversion of CHO to ANT must be attenuated. This step is catalyzed by ANS, which is competitively inhibited by TRP [42]. Inactivation of ANS was not carried out to avoid TRP auxotrophy and supplementation of TRP. An ANS variant was described in *P. taiwanensis* with the amino acid substitution P290S, which reduced the enzyme activity and thus increased phenol production from TYR [43]. An amino acid sequence alignment of ANS from *P.* *taiwanensis* and *C. glutamicum* revealed that this position is conserved in the ANS component I of *C. glutamicum* (TrpE-P304). Hence, the TrpE-P304S substitution was introduced into *C. glutamicum p*-CA3. The effect of the amino acid substitution on *p*-CA formation was examined by the cultivation of *C. glutamicum p*-CA3 TrpE-P304S (*C. glutamicum p*-CA4) in shake flasks (Fig. 4).

The amino acid substitution TrpE-P304S in *C. glutamicum p*-CA4 led to an increase in the product titer to 585 ± 12 mg/L (3.6 mM). More importantly, no accumulation of ANT was detectable, and growth was not affected, indicating that ANS activity was successfully modulated with the P304S mutation. Subsequently, to avoid any possible re-import of *p*-CA secreted into the culture supernatant, *phdT* encoding the phenylpropanoid permease PhdT involved in the import of phenylpropanoids was deleted to construct *C. glutamicum p*-CA4 ∆*phdT* (*C. glutamicum p*-CA5). However, the *phdT*-deficient strain and the parental strain performed almost identically in terms of growth and *p*-CA titers (Additional file 1: Fig. S2). This suggested that *phdT* deletion and thus the loss of the phenylpropanoid permease did not affect product formation. Nevertheless, *phdT* deletion could be beneficial for further strain construction, as less *p*-CA is likely to be re-imported by the *p*-CA-producing strain.

In order to identify further metabolic engineering targets, the culture supernatant of *C. glutamicum p*-CA5 was analyzed by HPLC. Besides *p*-CA and ANT, the central intermediate SA was identified as a major byproduct. Subsequently, efforts were made to reduce the accumulation of SA. The GTG translational start codon of *aroK* encoding shikimate kinase (SAK) was replaced by ATG (ATG-*aroK*). SAK catalyzes the conversion of SA to shikimate-3-phosphate (S3P) so that the presumed increase in AroK activity should reduce SA accumulation [44]. The *p*-CA titer of the resulting strain *C. glutamicum p*-CA5 ATG-*aroK* (*C. glutamicum p*-CA6) (Additional file 1: Fig. S3) increased from 517 ± 13.5 mg/L (3.2 mM) to 595 ± 14.2 mg/L (3.6 mM). Surprisingly, the accumulation of SA increased simultaneously by 33% to 276 ± 7.7 mg/L (1.7 mM). This suggested that the presumably faster conversion of SA to S3P also promoted the conversion of 3-dehydroshikimate (DHS) to SA [45]. To test the hypothesis that the conversion of SA to CHO represents a bottleneck, the carbon flux to SA was further increased by chromosomal integration of aroF*_EcCg_ encoding a feedback-resistant DAHP synthase from *E. coli.* The gene was integrated into the genome under the control of the constitutive *dapA* promotor variant A16 resulting in *C. glutamicum p*-CA6 IGR9::aroF*_EcCg_ (*C. glutamicum p*-CA7). The integration of aroF*_EcCg_ had a minor effect on the growth and the *p*-CA titer showed no significant difference compared to the control (Additional file 1: Fig. S4). GC-TOF MS analysis of the culture supernatants further supported the hypothesis that the conversion of SA is a bottleneck, reflected in DAHP, DHS, and SA accumulation along with minor amounts of protocatechuate (PCA). On the other hand, CHO synthesis could also be limited by the availability of the SA pathway precursors PEP and E4P.

Therefore, the pyruvate kinase gene *pyk* was deleted to increase the intracellular PEP availability by preventing the conversion of PEP to pyruvate [46]. The constructed strain *C. glutamicum p*-CA7 ∆*pyk* (*C. glutamicum p*-CA8) was cultivated in shake flasks and the effect of *pyk*-deficiency on *p*-CA production was investigated (Additional file 1: Fig. S5). The deletion of *pyk* resulted in no growth defect with glucose as the sole carbon source compared to the control with intact *pyk* gene as both strains reached an OD_600_ of 55 after 24 h (Additional file 1: Fig. S5). However, even though the growth of the *pyk*-deficient variant was not negatively affected, only a neglectable, but significant increase in product formation could be observed. Without pyruvate kinase activity, a *p*-CA titer of 551 ± 11.3 mg/L (3.4 mM) could be determined after 72 h of cultivation, which was only slightly higher compared to the parental strain (528 ± 21.5 mg/L (3.2 mM) *p*-CA). Since no increase in *p*-CA production could be determined by the deletion of *pyk*, the limitation in *p*-CA production might be the insufficient availability of E4P or the accumulating intermediates of the SA pathway preventing a further production increase.

Taken together, by inactivating competitive pathways, the introduction of feedback-resistant DAHP synthases, preventing byproduct formation, and increasing the carbon flux into the SA pathway, the *p*-CA titer could be increased by a factor of 7.3 compared to the starting strain.

### Limitation of inorganic phosphate to improve *p*-CA production parameters

**Fig. 5:**
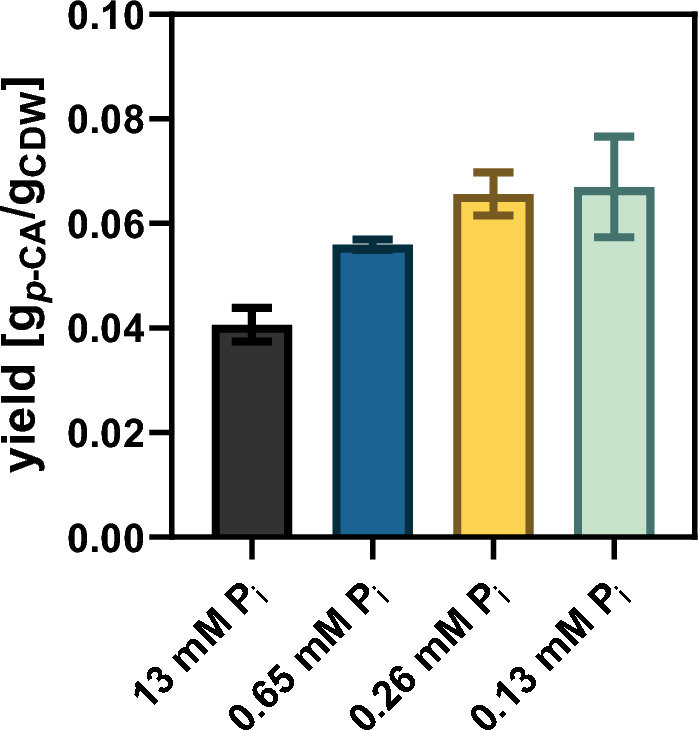
Specific *p*-CA production under non-limiting and P_i_-limiting conditions in defined CGXII medium. *C. glutamicum p*-CA8 pEKEx3-*aroH*_*Ec*_-*tal*_*FjCg*_ was cultivated in defined CGXII medium with varying P_i_ concentrations (13 mM (100%), 0.65 mM (5%), 0.26 mM (2%), 0.13 mM (1%)). The yield was calculated as the ratio of *p*-CA concentration in the culture supernatant and the cell dry weight (CDW) after 72 h of cultivation. The depicted data represent mean values and standard deviation of biological triplicates

In order to improve the *p*-CA production process, cultivation parameters were adjusted to divert the available carbon towards *p*-CA. In glucose-grown cells, growth is linearly dependent on the phosphate uptake rate [47]. Therefore, the amount of inorganic phosphate (P_i_) was limited (P_i_ concentrations: 13 mM (100%), 0.65 mM (5%), 0.26 mM (2%), or 0.13 mM (1%)) for the engineered *C.* *glutamicum p*-CA8 variant during cultivation on defined CGXII medium. This limitation aims at restricting the amount of carbon used for growth, leaving more for *p*-CA formation (Additional file 1: Fig. S6). By cultivating *C. glutamicum p*-CA8 under default conditions with 13 mM P_i_ (100%), the strain reached an OD_600_ of 66.2 ± 1.3 corresponding to a CDW of 21.6 ± 0.5 g/L after 24 h. Under this control condition, the highest *p*-CA titer achieved in this study was determined to be 661 ± 21.2 mg/L (4 mM) (0.017 g_*p*-CA_/g_glucose_). The reduction of the P_i_ concentration to 0.65 mM (5%) decreased the maximum biomass to an OD_600_ of 30.1 ± 1.6 (13.7 ± 0.6 g/L CDW), which was reached with a delay of 8 h compared to the control condition with 13 mM P_i_. Consequently, further P_i_ limitation caused a further restriction in growth. At the lowest P_i_ concentration of 0.13 mM (1%), an OD_600_ of 16.7 ± 0.7 was reached after 24 h. Despite the strongly impaired growth under P_i_-limited conditions, similar *p*-CA titers ranging from 566 ± 2.3 mg/L (3.4 mM, 5% P_i_), 607 ± 8.2 mg/L (3.7 mM, 2% P_i_), and 567 ± 17 mg/L (3.5 mM, 1% P_i_) were achieved compared to standard P_i_-concentration. Subsequently, the specific *p*-CA production was calculated to illustrate the correlation between decreased P_i_ concentration and production, showing that the yield (g_*p*-CA_/g_CDW_) increased with decreasing P_i_ concentration from 0.041 ± 0.003 to 0.067 ± 0.009 with 1% P_i_, which corresponds to an increase of 39% (Fig. 5). In addition, the volumetric productivity was calculated after 72 h to include the different rates of product formation at different P_i_ concentrations. Interestingly, the highest volumetric productivity of 9.2 ± 0.3 mg/L/h was determined for the standard condition (13 mM P_i_), followed by 7.9 ± 0.03 mg/L/h (0.65 mM P_i_), 8.4 ± 0.1 mg/L/h (0.26 mM P_i_) and 7.9 ± 0.2 mg/L/h (0.13 mM P_i_). This indicated that the volumetric productivity is inversely related to the product yield as the P_i_ concentration decreases.

### Co-cultivation for microbial PNP production from glucose

**Fig. 6:**
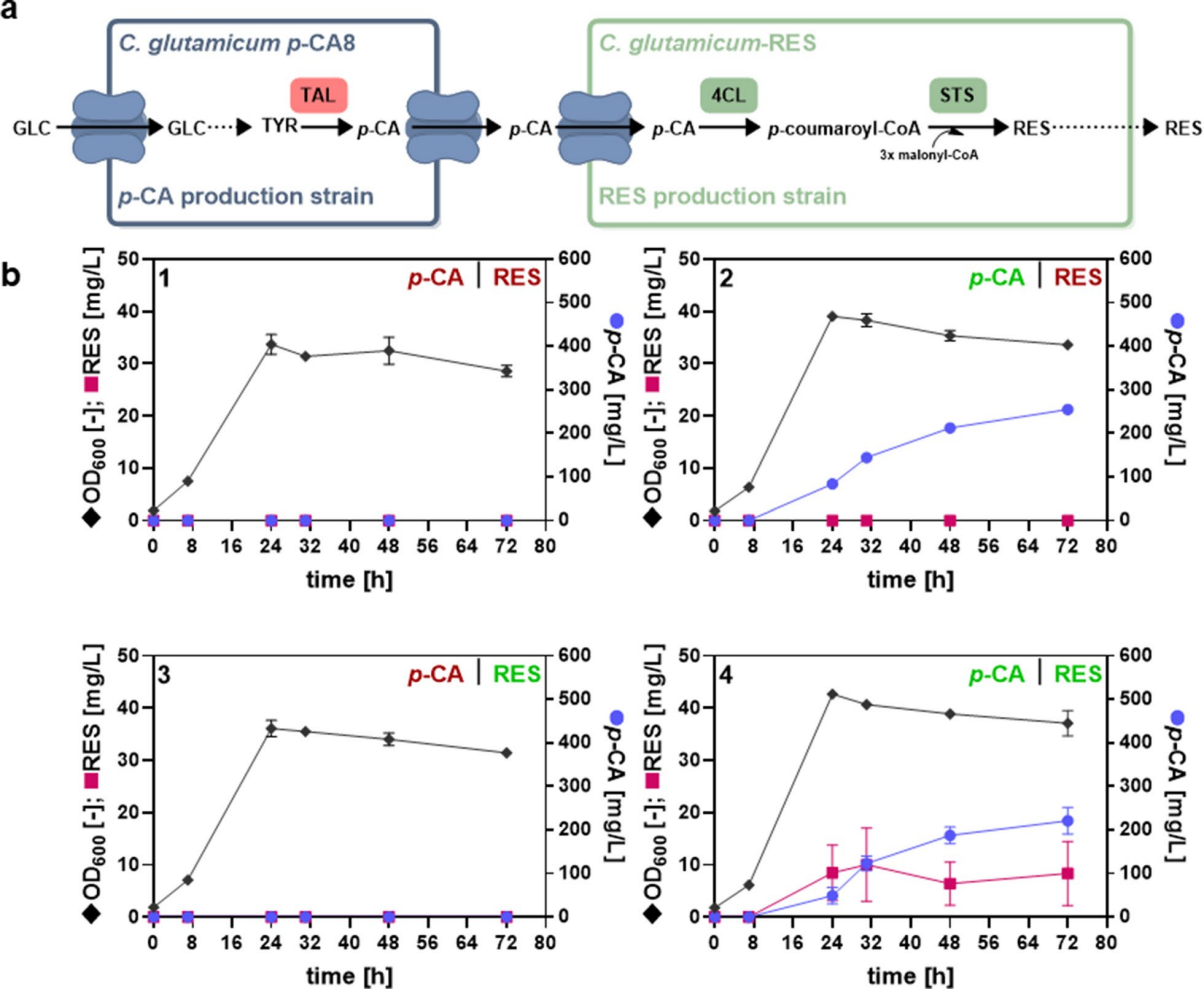
Co-cultivation of *C. glutamicum* strains optimized for *p*-CA and RES production. **a** The *p*-CA production strain *C. glutamicum p*-CA8 serves as a module providing the RES-precursor *p*-CA using glucose as a carbon source, whereas the RES-producing strain *C. glutamicum*-RES takes up *p*-CA for the synthesis of RES. *4CL* 4-coumarate:CoA ligase, *p-CA* *p*-coumaric acid, *RES* resveratrol, *STS* stilbene synthase, *TYR* tyrosine, *TAL* tyrosine ammonia-lyase. **b** Production of RES in a co-cultivation system. The joint growth (OD_600_, diamonds), *p*-CA (circles), and RES titers (squares) of the two strains *C. glutamicum p*-CA8 and *C. glutamicum*-RES are depicted. An inoculation ratio of 1:1 was chosen for the cultivation. The green font color indicates that the *p*-CA production strain was able to form *p*-CA, while the RES production strain was able to produce RES. This was achieved by the presence of the corresponding production plasmid pEKEx3-*aroH*_*Ec*_-*tal*_*FjCg*_ (*p*-CA) or pMKEx2-*sts*_*Ah*_-*4cl*_*Pc*_ (RES). The red font color indicates the inability to form *p*-CA or RES, which was evoked by the presence of the corresponding empty plasmids. As controls, both strains were cultivated that could form (1) neither *p*-CA nor RES, (2) those that could form only *p*-CA (3) or RES, (4) and those that could form both *p*-CA and RES. The data represent mean values and standard deviation of biological triplicates

Microbial production of PNPs such as RES, naringenin, or raspberry ketone with *C. glutamicum* typically took advantage of the supplementation of 5 mM *p*-CA dissolved in DMSO as a precursor molecule [32, 48, 49]. The strain constructed in the course of this study offers the possibility of co-cultivations to enable the production of valuable PNPs from glucose, which would no longer be dependent on the costly supplementation of *p*-CA. In principle, the *p*-CA-providing strain could be combined with any strain engineered for the synthesis of *p*-CA-derived products. As a proof-of-concept, the biotechnologically interesting stilbenoid RES was selected for microbial production in a co-cultivation set-up taking advantage of the constructed *p*-CA production strain *C. glutamicum p*-CA8. For this purpose, the RES production strain *C. glutamicum* DelAro^4^-*4cl*_*Pc*_ C5 *mufasO*_*BCD1*_ P_O6_-*iolT1* Δ*pyc* pMKEx2-*sts*_*Ah*_-*4cl*_*Pc*_ (*C. glutamicum*-RES) was used, which was engineered for increased intracellular availability of malonyl-CoA required for the synthesis of most plant polyphenols [31, 32]. In a co-cultivation process, *p*-CA synthesized by *C. glutamicum p*-CA8 would be exported into the culture supernatant and taken up by *C. glutamicum*-RES, which still harbors the phenylpropanoid-permease PhdT for *p*-CA import (Fig. 6a) [37]. *C. glutamicum*-RES is capable of CoA-activating *p*-CA by a 4-coumarate: CoA ligase (4CL). Subsequently, *p*-coumaryl-CoA would be converted to RES by a stilbene synthase (STS)-catalyzed condensation with three molecules of malonyl-CoA. The required genes *sts*_*Ah*_ and *4cl*_*Pc*_ are located on the production plasmid pMKEx2 and code for STS from *Arachis hypogea* and 4CL from *Petroselinum crispum*. In order to enable RES production from glucose, both strains were co-cultivated in shake flasks with glucose as the sole carbon source (Fig. 6b). As controls, strain combinations were co-cultivated, which can only accumulate *p*-CA, RES, or neither of these two compounds. This was realized by the presence or absence of the respective empty plasmid.

The control experiments indicated that neither *p*-CA nor RES could be produced from glucose in the absence of a strain carrying the *p*-CA- or RES production plasmid (Fig. 6b, 1–3). If only *p*-CA could be formed, no RES was produced, if the RES production strain carried the empty plasmid. If, on the other hand, *p*-CA could not be formed, RES synthesis failed due to the absence of the precursor. In the presence of two suitable strains, a maximum RES titer of 10 ± 7 mg/L (0.044 mM) could be determined after 32 h, demonstrating that RES production by *C. glutamicum*-RES was achieved from *p*-CA provided by *C. glutamicum p*-CA8. RES-synthesis ceased after 32 h due to the exhaustion of malonyl-CoA, whose formation is strictly coupled to cell growth [50]. The high standard deviation of the RES titer indicated that RES production differed strongly between each replicate, which might be caused by minor differences in the initial ratios of the *p*-CA and RES-producing strains, exacerbated by different exponential growth rates in the co-culture. This assumption was supported by 222 ± 30.7 mg/L (1.4 mM) *p*-CA determined in the culture supernatant of the RES-producing co-culture, which was not taken up and converted to RES by *C.* *glutamicum*-RES. To test if an imbalanced ratio of both production strains hindered efficient *p*-CA to RES conversion, the individual growth rates of both strains were determined (Additional file 1: Fig. S7). The *p*-CA production strain entered the exponential growth phase after 6 h and was characterized by a growth rate of 0.13 h^−1^. In comparison, the RES production strain showed a prolonged lag phase of 10 h and a reduced growth rate of 0.10 h^−1^, which resulted in a joint growth rate of 0.12 h^−1^ determined for the co-cultivated strains. The surplus of *p*-CA measured previously might be associated with the higher growth rate of the *p*-CA producer. Thus, the inoculation ratios of both strains were adjusted with the aim to prevent *p*-CA accumulation and increase RES production. The inoculation OD_600_ of *C. glutamicum p*-CA8 to *C. glutamicum*-RES were set to 1:1, 0.75:1.25, 0.5:1.5, and 0.2:1.8 and the effect on *p*-CA and RES production was examined (Additional file 1: Fig. S8). The inoculum reduction of the *p*-CA producer resulted in a decrease of the *p*-CA titer from 157 ± 4.9 mg/L (0.9 mM) (1:1 ratio) to 11.1 ± 0.8 mg/L (0.07 mM) (0.2:1.8 ratio). However, RES production decreased simultaneously from 31.2 ± 3.2 mg/L (0.1 mM) (1:1 ratio) to 3.9 ± 2.1 (0.02 mM) (0.2:1.8 ratio), indicating that RES production could not be improved by increasing the inoculum of the RES production strain with a lower growth rate than the *p*-CA producer. Possibly, RES production is limited by the *p*-CA uptake rate, which is gradient-driven through the permease meaning that higher concentrations increase uptake. This notwithstanding, the *p*-CA-producer constructed here could be successfully utilized in a co-cultivation setup for the synthesis of RES from glucose without supplementation of any precursors. These results indicate that modular co-cultivation of *C. glutamicum* is a suitable strategy to divide intricate heterologous pathways between specialized strains.

## Discussion

In this study, *C. glutamicum* strains for the microbial synthesis of the phenylpropanoid *p*-CA were constructed. Initially, cytotoxicity tests with *p*-CA were performed since *p*-CA as free acid is known to disrupt bacterial cell membranes and intercalate into genomic DNA [51]. These experiments showed that *p*-CA concentrations exceeding 6.1 mM prolonged the lag phase of the *C. glutamicum* ATCC13032 wild type compared to the DMSO control. In the context of a previous study in which *C.* *glutamicum* was genetically modified to produce RES, the growth of a highly-engineered production strain was already impaired at lower concentrations of only 1 mM *p*-CA [48]. Here, it was also observed that the strain variant *C. glutamicum p*-CA1 exhibited a lower growth rate than the wild type and showed an overall reduced biomass formation with increasing *p*-CA concentrations. This could be due to the absence of catabolic pathways for aromatic compounds including the *phd* pathway for phenylpropanoids degradation, which would break down cytotoxic *p*-CA to non-toxic TCA-cycle intermediates [37, 48]. However, despite these modifications*, C. glutamicum* is more robust than most other microorganisms, since the minimum inhibitory concentration of *p*-CA determined for other gram-positive bacteria including *Streptococcus pneumonia, Staphylococcus aureus,* and *B. subtilis* was only 20 µg/mL (0.12 mM), and 80 µg/mL (0.49 mM) for the gram-negative bacterium *E. coli* [51]. Only an engineered *P.* *putida* variant with tolerance against high *p*-CA concentration is available, which was obtained by adaptive laboratory evolution [52].

During the construction of the *p*-CA-producing *C.* *glutamicum* variants, the translation efficiency of the prephenate dehydratase gene *pheA* was reduced by replacing the canonical ATG start codon with GTG, avoiding PHE auxotrophy. A similar strategy was pursued for TYR production with *C. glutamicum* [53]. In this case, the start codon was changed to the least preferred TTG, which severely reduced the expression of *pheA* and rendered the expensive supplementation of 2 mM PHE necessary to restore growth. In addition, a PHE-auxotrophic *P. putida* strain was constructed in the context of *p*-CA production to reduce byproducts and control cell growth and product formation via PHE supplementation [23]. With this strategy, the product titer was increased by a factor of four, but it remains uncertain whether the costs of PHE supplementation can be economical in possible future applications. A significant increase in the product titer was achieved by implementing a feedback-resistant endogenous DAHP-synthase AroF. However, a concomitant accumulation of ANT was observed, which is known to have negative impacts on growth as ANT interferes with biomass formation and impairs other cellular processes by the inhibition of specific enzymes [54, 55]. The formation of ANT was reduced to undetectable amounts by the amino acid substitution TrpE-P304S, presumably reducing ANS activity. This mutation was previously introduced in the strain *P. taiwanensis* VLB120, which was one of the decisive factors for high phenol production [43]. The amino acid substitution TrpE-P304 is conserved among *C. glutamicum* and *P. taiwanensis* and downregulation of ANT synthesis is a suitable strategy for the production of *p*-CA and other CHO-derived aromatics in *C. glutamicum* without causing TRP auxotrophy. In *P. taiwanensis*, the mutation in TrpE led to a major increase in aromatics production, whereas overexpression of feedback-resistant DAHPS genes had only a minor effect on product synthesis [43]. In this study, the exact opposite was observed for *C. glutamicum*, highlighting the differentially regulated synthesis of aromatic compounds in these two organisms.

GC-TOF MS analysis of culture supernatants was performed to detect the accumulation of other undesired byproducts. These experiments showed that predominantly SA pathway intermediates such as DAHP, DHS, and SA accumulated in the engineered variants. With the aim to reduce SA accumulation, the GTG-start codon of the SAK gene *aroK* was replaced by ATG to increase the translation efficiency. Surprisingly, the SA accumulation increased, which might be due to the mode of regulation of the shikimate dehydrogenase (SDH) catalyzing the reduction of DHS to SA. The SDH-activity is negatively feedback-regulated by the presence of SA, but SA is also known to have a negative effect on the transcription of the SDH-gene *aroE* [45]. According to this, inhibition of SDH is reduced by increased SA phosphorylation by SAK resulting in increased conversion of DHS to SA. Accumulation of DHS and SA could also be related to the activity of SAK, which is feedback-inhibited by SA [56]. To circumvent these complex layers of regulation, a codon-optimized gene encoding a feedback-resistant SAK from *Methanocaldococcus janaschii* could be functionally implemented into the metabolism of *C.* *glutamicum*, a strategy which was already successfully pursued in the context of 4-hydroxybenzoate production with this bacterium [56]. Another alternative to reduce SA accumulation might be the utilization of SAK II (AroL) from *E. coli*, which is characterized by a reduced K_m_ value of 0.2 mM SA compared to AroK with 20 mM of the same organism [57]. Heterologous expression of *aroL* from *E.* *coli* in *S. cerevisiae* was described to have a positive effect on *p*-CA production [21]. Moreover, in addition to the SAK, overexpression of *ydib*-encoded SDH from *E. coli* was associated with an increase in the TYR titer [58]. Hence, the role of SDH in *p*-CA production could be elucidated in future experiments. Furthermore, the SA pathway genes *aroB*, *aroD*, *aroE,* and *aroG* are not organized as an operon in *C. glutamicum*, which hampers their genetic manipulation. Another approach would therefore be to adjust the ribosome binding site (RBS) of each gene to the consensus sequence of *C. glutamicum* to improve the translation of the *aro* genes. The construction of plasmid-based continuous genetic modules of the SA pathway with tailored RBS libraries for each *aro* gene was shown to allow for an increased SA synthesis from 80 mg/L to 4.3 g/L with *C. glutamicum* [59]. The deletion of *pyk* did not result in improved *p*-CA production through increased PEP availability. Hence, strategies for increased E4P availability could also be applied, since a balanced ratio of both SA pathway precursors is necessary for increased synthesis of aromatics. In this context, homologous overexpression of the transketolase-encoding *tkt* gene from *C. glutamicum* improved the product titer of TRP previously [60]. Other studies in *E. coli* focused on the overexpression of the transaldolase gene *talB* for DAHP production or the deletion of phosphoglucose isomerase gene *pgi*, which rerouted the metabolic flux from glucose to the pentose phosphate pathway [61, 62].

The constructed *p*-CA production strain *C. glutamicum p*-CA8 was subsequently used as a module to provide *p*-CA in co-cultivations to synthesize the *p*-CA-derived RES directly from glucose. The co-cultivation thus allowed the metabolic burden generated by heterologous gene expression to be partitioned between two strains. In addition, the feedback inhibition of the TAL enzyme towards *p*-coumaroyl-CoA is circumvented in such co-cultivations since CoA-activation of *p*-CA occurs only in the RES-producing *C.* *glutamicum* variant [63]. In the past, 12 mg/L RES from glucose were produced with a single *C. glutamicum* variant engineered for RES production from glucose without supplementation of any precursors [31]. Co-cultivation of two *C. glutamicum* strains as presented in this study, allowed for a final RES titer of 31.2 mg/L, which was markedly higher compared to the monoculture of a *C.* *glutamicum* variant bearing the full metabolic burden of RES synthesis from glucose. Previously, two *E. coli* strains were co-cultivated with a similar setup – one *p*-CA- and one RES-producing variant [64]. In these experiments, 22.6 mg/L RES was produced from glycerol [64]. Furthermore, a *p*-CA-producing variant of *E. coli* and a RES-producing yeast strain were co-cultivated allowing for the accumulation of 28.5 mg/L RES from glucose [38]. In the case of the co-cultivation setup presented in this study, cultivation parameters such as the inoculation ratios would have to be further adjusted, since the *p*-CA-producing variant is characterized by a higher growth rate compared to the RES-producing strain. This imbalance was reflected in an excess of *p*-CA in the supernatant in all performed co-cultivation experiments. In their work with *C. glutamicum* strains on riboflavin production, Pérez-García and coworkers were able to show that the inoculation timing of the second strain can influence product synthesis and yield. There, the riboflavin yield could be increased from 0.22 mg/g to 0.39 mg/g by inoculation in the stationary growth phase [65]. Two riboflavin-producing *C. glutamicum* strains were used that were adapted to metabolize mannose or xylose by plasmid-based expression of *manA* and *xylAB*. In this context, the utilization of different carbon sources is a suitable strategy to prevent one strain from overgrowing the other, which highlights the potential of a mixed substrate for the co-cultivation established here [65]. Moreover, it is possible to create a mutual dependence of both strains via the introduction of auxotrophies to generate a stable community. This was previously achieved for the co-cultivation of two *C. glutamicum* strains, where each strain provided a different amino acid to complement the auxotrophy of the respective other variant [66]. However, these cross-feeding strategies typically lead to a higher metabolic burden, which co-cultivations are actually supposed to counteract. In order to engineer mutual dependence and investigate complex interactions in co-cultivations in more detail, microfluidic cultivation platforms can be used [67]. The strategies presented here might be useful starting points for the development of more fine-tuned co-cultivation processes for PNP production with *C. glutamicum* in the future. This appears worthwhile, when considering that the phenylpropanoid *p*-CA is an important precursor for a broad range of other PNPs, such as flavonoids or flavoring phenylbutanoids. Hence, the *p*-CA-producing variant constructed in this study can serve as *p*-CA-module in co-cultivations with other microbial variants designed to synthesize *p*-CA-derived natural products.

## Conclusions

In this study, we enabled the microbial de novo production of the versatile phenolic compound *p*-CA with *C. glutamicum*. Key to improving product formation was the utilization of genome editing techniques to modulate the SA pathway and the branched aromatic amino acid metabolism. The *p*-CA producer constructed in this study not only expands the aromatic product portfolio of *C. glutamicum*, but also lays the foundation for the sustainable synthesis of polymer building blocks for biodegradable plastics and the production of plant secondary metabolites.

## Methods

### Bacterial strains, media, and cultivation conditions

Strains and plasmids utilized in this study are shown in Table. 1 and oligonucleotides used are listed in Additional file 1: Table S1. *C. glutamicum* strains were cultivated aerobically at 30 ℃ in brain heart infusion (BHI) medium (Difco Laboratories, Detroit, USA) or in defined CGXII medium supplemented with 4% (w/v) glucose as sole carbon and energy source [68]. To establish inorganic phosphate (P_i_) limiting conditions, a 200 × stock solution of KH_2_PO_4_ and K_2_HPO_4_ (5 g KH_2_PO_4_ and 5 g K_2_HPO_4_ dissolved in 25 mL ddH_2_O) was prepared, and 5 mL were added to 1 L CGXII medium to a final P_i_ concentration of 13 mM (100%). This stock solution was diluted with ddH_2_0 to achieve final P_i_ concentrations of 0.65 mM (5%), 0.26 mM (2%), or 0.13 mM (1%).

**Table 1:** Strains and plasmids used in this study

Strain or plasmid	Characteristics	Source
C. glutamicum strains		
DelAro^4^-*4cl*_*Pc*_ C5 muf*asO*_*BCD1*_ P_O6_-*iolT1* ∆*pyc* (M-CoA)	Prophage-free derivative of ATCC13032 with an in-frame deletion of cg0344-47, cg0503, cg2625-40, and cg1226. Chromosomal integration of the codon-optimized gene variant *4cl*_*PcCg*_ encoding 4-coumarate: CoA ligase from parsley (*Petroselinum crispum*) (Δcg0344-47:PT7-*4cl*_*PcCg*_) Exchange of the native promotor of the citrate synthase gene *gltA* by *dapA* promotor variant C5. Mutated *fasO* binding site upstream of *accBC* and *accD1*. Two point mutations in the promotor of the inositol transporter gene *iolT1* abolish repression of *iolT1* by IolR. In-frame deletion of *pyc* (cg0791) encoding pyruvate carboxylase	[50]
DelAro^5^ C7 P_O6_-*iolT1* (*p*-CA1)	Prophage-free derivative of ATCC13032 with in-frame deletions of cg0344-cg0347, cg2625-cg2640, cg1226, cg502, and cg3349-cg3354 (DelAro^5^). Exchange of the native promotor of the citrate synthase gene *gltA* by *dapA* promotor variant C7. Two point mutations in the promotor of the inositol transporter gene *iolT1* abolish repression of *iolT1* by IolR	[35]
* p*-CA1 ∆*phdA* (*p*-CA2)	*p*-CA1 derivative with an in-frame deletion of *phdA* (cg0341) encoding an endogenous acyl:CoA ligase	This work
* p*-CA2 GTG-*pheA*	*p*-CA2 derivative with start codon replacement (ATG→GTG) of *pheA* (cg3207) encoding prephenate dehydratase	This work
* p*-CA2 AroF-S188C	*p*-CA2 derivative harboring the mutated DAHP synthase AroF carrying the amino acid substitution S188C	This work
* p*-CA2 GTG-*pheA* AroF-S188C (*p*-CA3)	*p*-CA2 derivative with start codon replacement (ATG→GTG) of *pheA* (cg3207) encoding prephenate dehydratase and harboring the mutated DAHP synthase AroF carrying the amino acid substitution S188C	This work
* p*-CA3 TrpE-P304S (*p*-CA4)	*p*-CA3 derivative harboring the mutated anthranilate synthase component I TrpE carrying the amino acid substitution P304S	This work
* p*-CA4 ∆*phdT* (*p*-CA5)	*p*-CA4 derivative with an in-frame deletion of *phdT* (cg0340) encoding the phenylpropanoid transporter PhdT	This work
* p*-CA5 ATG-*aroK* (*p*-CA6)	*p*-CA5 derivative with start codon replacement (GTG→ATG) of *aroK* (cg1828) encoding shikimate kinase	This work
p-CA6 IGR9::aroF*_EcCg_ (*p*-CA7)	*p*-CA6 derivative with genomic integration of aroF*_EcCg_ encoding AroF* from *E. coli* (codon-optimized gene) under control of the *dapA* promotor variant A16	This work
* p*-CA7 ∆*pyk* (*p*-CA8)	*p*-CA7 derivative with an in-frame deletion of *pyk* (cg2291) encoding pyruvate kinase	This work
*E. coli* strains		
* E. coli* DH5α	F^–^Φ80*lacZ*ΔM15 Δ(*lacZYA*- *argF*)U169 *recA1 endA1 hsdR17* (rK-, mK+) *phoA supE44λ- thi-1 gyrA96 relA1*	Invitrogen (Karlsruhe, Germany)
Plasmids		
pMKEx2	*kan*^r^; *E. coli*-*C. glutamicum* shuttle vector (*lacI*, P_T7_, lacO1, pHM1519 ori_*Cg*_; pACYC177 ori_*Ec*_)	[70]
pMKEx2-*sts*_*AhCg*_-*4cl*_*PcCg*_	pMKEx2 derivative for expression of *sts*_*AhCg*_ and *4cl*_*PcCg*_ (codon-optimized genes) encoding stilbene synthase from *Arachis hypogaea* and 4-coumarate:CoA ligase from *Petroselinum crispum*	[31]
pEKEx3	*spec*^r^; *E. coli*-*C. glutamicum* shuttle vector (*lacI*, P_*tac*_, lacO1, pBL1ori_*Cg*_; pUCori_*Ec*_)	[72]
pEKEx3-*aroH*_*Ec*_-*tal*_*FjCg*_	pEKEx3 derivative for expression of *aroH*_*Ec*_ encoding DAHP synthase from *E. coli* and *tal*_*FjCg*_ (codon-optimized gene) encoding tyrosine ammonia-lyase from *Flavobacterium johnsoniae*	[31]
pK19*mobsacB*	*kan*^*r*^*;* vector for allelic exchange in *C. glutamicum* (pK18 oriVEc *sacB* lacZα)	[73]
pK19*mobsacB*-∆*phdA*	pK19*mobsacB* derivative for in-frame deletion of *phdA*	[37]
pK19*mobsacB*-GTG-*pheA*	pK19*mobsacB* derivative for the start-codon replacement of *pheA* (ATG→GTG)	This work
pK19*mobsacB*-AroF-S188C	pK19*mobsacB* derivative for site-directed mutagenesis of *aroF* encoding DAHP synthase from *C. glutamicum* resulting in the amino acid substitution S188C	This work
pK19*mobsacB*-TrpE-P304S	pK19*mobsacB* derivative for site-directed mutagenesis of *trpE* encoding anthranilate synthase component I from *C. glutamicum* resulting in the amino acid substitution P304S	This work
pK19*mobsacB*-∆*phdT*	pK19*mobsacB* derivative for in-frame deletion of *phdT*	[37]
pK19*mobsacB*-ATG*-aroK*	pK19*mobsacB* derivative for the start-codon replacement of *aroK* (GTG→ATG)	Mutz et al.(in preparation)
pK19*mobsacB*-IGR9::P*dapA*-A16-aroF*_EcCg_	pK19*mobsacB*-IGR9 derivative for genomic integration of aroF*_EcCg_, whose gene expression is under control of the *dapA* promotor variant A16	Mutz et al.(in preparation)
pK19*mobsacB*-∆*pyk*	pK19*mobsacB* derivative for in-frame deletion of *pyk*	[74]

For the cultivation of *C. glutamicum*, test tubes with 5 mL BHI medium supplemented with the appropriate antibiotic were inoculated from a single colony grown on a BHI agar plate. These first precultures were grown for 8 h on a rotary shaker at 170 rpm. The precultures were used to inoculate second precultures, harboring 50 mL defined CGXII medium supplemented with 4% (w/v) glucose in 500 mL baffled flasks. These second precultures were cultivated overnight on a rotary shaker at 130 rpm. Bacterial growth was tracked by measuring the optical cell density at 600 nm (OD_600_) at defined time points throughout the cultivation. CGXII main cultures were inoculated to an initial OD_600_ of 1 from the second precultures. For co-cultivation experiments, both the *p*-CA and PNPs producing strain were inoculated to an initial OD_600_ of 1 corresponding to a total OD_600_ of 2. If indicated, the inoculum ratios (*p*-CA to PNP producing strain) were adjusted from 1:1 to 0.75:1.25, 0.5:1.5, 0.5:0.5, or 0.2:1.8. The chromosomally encoded T7 RNA polymerase gene enables IPTG-inducible gene expression from the T7 promotor [69, 70]. Thus, for *p*-CA production, plasmid-based gene expression was induced by the addition of 1 mM IPTG 1 h after inoculation. If two plasmids were present in the production strains, the induction strength was reduced to 20 µM IPTG as higher IPTG concentrations resulted in impaired growth and production. At defined time points, 1 mL of the culture broth was sampled, centrifuged at 13,300 rpm for 20 min and the culture supernatant was stored at – 20 ℃ until HPLC analysis. For the determination of the cell dry weight (CDW), another sample was withdrawn from the cultivation broth. The samples were centrifuged at 13,300 rpm for 20 min and the supernatant was discarded. Subsequently, the cell pellets were completely dried by centrifugation under vacuum at 60 °C for 2 h using the rotating evaporator *Eppendorf Concentrator Plus* (Eppendorf, Hamburg, Germany).

Cytotoxicity of *p*-CA was investigated by the cultivation of *C. glutamicum* at 30 ℃, 900 rpm, and a humidity of 85% for 24 h in 48-well Flowerplates containing 800 µL defined CGXII medium with 2% (w/v) glucose inoculated to an initial OD_600_ of 1 by using the BioLector microbioreactor (m2p-labs, Baesweiler, Germany). Increasing concentrations of *p*-CA (final concentration of 0.125 g/L (0.76 mM), 0.25 g/L (2.5 mM), 0.5 g/L (3 mM), 0.75 g/L (4.6 mM), 1 g/L (6.1 mM), 1.5 g/L (9.1 mM) and 2 g/L (12.2 mM) *p*-CA) were added to the medium. For this, an 80 g/L (487.7 mM) *p*-CA stock solution, dissolved in DMSO was prepared. This stock solution was diluted with DMSO to adjust all desired *p*-CA concentrations and DMSO without *p*-CA was added as a control. Growth was tracked by measuring the backscattered light intensity (620 nm, gain 10).

Plasmid construction and cloning were performed using *E. coli* DH5α. Cultivation of *E. coli* strains was routinely performed in Lysogeny Broth (LB) medium (10 g/L tryptone, 10 g/L NaCl, 5 g/L yeast extract) at 37 ℃ [71]. Where necessary, kanamycin (*E. coli*: 50 µg/mL; *C. glutamicum*: 25 µg/mL for cultivations and 15 µg/mL for colony selection after transformation) and/or spectinomycin (100 µg/mL for both, *E. coli* and *C. glutamicum*) was added to agar plates and liquid media.

### Plasmid and strain construction

PCR, gel electrophoresis, DNA restriction and ligation, and further standard cloning work were performed as described previously [75]. Genes and chromosomal fragments required for plasmid constructions were amplified by PCR using genomic *C. glutamicum* DNA or whole cells as templates and oligonucleotides shown in Additional file 1: Table S1. Plasmids for homologous recombination or expression of homologous and heterologous genes were constructed by Gibson assembly [76]. Gene deletions, the introduction of point mutations for up or down-regulation of metabolic pathways, or genomic integration of whole genes in the genome of *C. glutamicum* were performed by using a two-step homologous recombination method described previously [73, 77]. Colony PCR, restriction analysis and DNA sequencing performed at Eurofins MWG Operon (Ebersberg, Germany) verified the construction of plasmids and the identity of recombinant *C. glutamicum* strains. Transformation of *C. glutamicum* with constructed plasmids was performed by electroporation [78].

### HPLC and GC-TOF MS analysis

The detection of *p*-CA and ANT in the culture supernatant was performed with a high-performance-liquid-chromatography (HPLC) 1260 Infinity II System equipped with a 1260 Infinity II Diode Array Detector (DAD) (Agilent Technologies, Waldbronn, Germany). Samples of the culture supernatant were centrifuged at 13,300 rpm for 20 min to remove cells and precipitated media components. The cell-free supernatants were transferred into fresh reaction vessels and stored at – 20 ℃ until HPLC analysis. An authentic *p*-CA standard (1 g/L dissolved in acetonitrile) was diluted 1:1 to 31.25 mg/L. For the quantification of ANT, a 10 mM standard was prepared and diluted 1:1 to 31.25 mM. The isocratic separation of *p*-CA and ANT was performed using an *Agilent InfinityLab Poroshell* 120 2.7 µM EC-C_18_ column (3.0 × 150 mm; Agilent Technologies, Waldbronn, Germany) with an *Agilent InfinityLab Poroshell* 2.7 µM EC-C_18_ pre-column (3 × 5 mm; Agilent Technologies, Waldbronn, Germany) at a flow rate of 0.35 mL/min at 40 ℃. The mobile phase consisted of 0.1% (v/v) acetic acid (solvent A, 80%) and acetonitrile supplemented with 0.1% (v/v) acetic acid (solvent B, 20%). The absorption of *p*-CA was measured at 310 nm and of ANT at 220 nm.

For the isocratic separation of organic acids and sugars a Rezex ROA-Organic Acid H^+^ column 8 µm (300 × 7.8 mm; Phenomenex, Torrance, California, USA) and the pre-column Security Guard HPLC Guard Cartridge system Carbo-H 4 x (4 × 3 mm; Phenomenex, Torrance, California, USA) was applied at 80 ℃. For elution from the column, 5 mM H_2_SO_4_ was used as the mobile phase at a flow rate of 0.3 mL/min. An authentic SA standard was prepared (10 mM, dissolved in ddH_2_O) and diluted to 0.3125 mM. A glucose standard of 100 mM was prepared and diluted to 3.125 mM. The concentration of SA was determined by measuring the absorption at 220 nm, while glucose was detected by the refractive index detector (RID).

For the quantification of RES, cultivation samples were extracted from the cultivation broth with ethyl acetate. 1 mL of ethyl acetate was added to 1 mL of culture broth and the samples were incubated in a thermomixer (Eppendorf, Hamburg, Germany) at 1,400 rpm and 23 ℃ for 15 min. Subsequently, samples were centrifuged at 13,300 rpm for 7 min. The upper organic phases were transferred to solvent-resistant deep-well plates and evaporated to dryness. The pellets were dissolved in the same volume of acetonitrile and analyzed by an ultrahigh-performance LC (uHPLC) 1290 Infinity System equipped with a 6130 Quandrupol LC–MS System (Agilent, Waldbronn, Germany) as described previously [31]. Briefly, Kinetex 1.7 µm C_18_ 100 Å pore size (2.1 × 50 mm, 1.7 µm; Phenomenex, Torrance, California, USA) column with the Security Guard ULTRA Cartridge C_18_ (2.1 mm; Phenomenex, Torrance, California, USA) pre-column was used at 50 ℃ for separation. The mobile phase (0.1% (v/v) acetic acid (solvent A); acetonitrile supplemented with 0.1% (v/v) acetic acid (solvent B)) was pumped at a flow rate of 0.5 mL/mL. For elution, a gradient was applied in which the proportion of solvent B was gradually increased (minute 0–6: 10–30%, minute 6–7: 30–50%, min 7–8: 50–100%, and min 8–8.5: 100–10%). The mass spectrometer (MS) was used in the electrospray ionization (ESI) mode. An authentic RES standard (250 mM dissolved in acetonitrile) was prepared and diluted with ddH2O to 3.9 mM. Benzoic acid was added as an internal standard at a final concentration of 100 mg/L. The ratio of the RES signal and internal standard was used for the calculation of the RES concentration. Area values of integrated signals were linear up to metabolite concentration of 500 mg/L *p*-CA, 1 g/L RES, 5 mM ANT, 10 mM SA, and 100 mM glucose.

Gas chromatography-time-of-flight (GC-TOF) mass spectrometry (MS) was performed for metabolite identification in culture supernatants using an Agilent 8890N double SSL gas chromatograph (Agilent, Waldbronn, Germany), which was equipped with a L-PAL3-S15 liquid autosampler coupled to a LECO GCxGC HRT+ 4D high-resolution TOF MS (LECO, Mönchengladbach, Germany). Typically, sample preparation, two-step derivatization of the samples, and MS data acquisition were performed as described previously [79]. Peak identification of known and unknown metabolites was performed as described before [80].

### Supplementary Information

**Additional file 1: Table S1.** Oligonucleotides used in this study. **Figure S1.** Impact of (ATG→GTG) start codon replacement of *pheA* and introduction of the point mutation into AroF (AroF-S188C) on growth and metabolite accumulation. Growth (OD_600_) (diamonds), *p*-CA titer (circles), and ANT concentration (squares) of (**a**) *C. glutamicum* *p*-CA2, (**b**) *C. glutamicum* *p*-CA2 GTG-*pheA*, (**c**) *C. glutamicum* *p*-CA2 AroF-S188C and (**d**) *C. glutamicum* *p*-CA2 GTG-*pheA* AroF-S188C (*p*-CA3). All strains harbor the expression plasmid pEKEx3-*aroH*_*Ec*_-*tal*_*FjCg*_ for *p*-CA production. The depicted data represent mean values and standard deviation of biological triplicates. **Figure S2.** Effect of an in-frame deletion of *phdT* on *p*-CA production. (**a**) Growth (OD_600_) and (**b**) *p*-CA titer of *C. glutamicum* *p*-CA4 (control, circles) and *C. glutamicum* *p*-CA4 ∆*phdT* (*p*-CA5). (squares). Both strains harbor the expression plasmid pEKEx3-*aroH*_*Ec*_-*tal*_*FjCg*_ for *p*-CA production. The depicted data represent mean values and standard deviation of biological triplicates. **Figure S3.** Effect of (GTG→ATG) start codon replacement of *aroK* encoding SAK on *p*-CA titer and SA accumulation. Growth (OD_600_, diamonds), *p*-CA (circles), and SA titer (squares) of (**a**) *C. glutamicum* *p*-CA5 (control) and (**b**) *C. glutamicum* *p*-CA5 ATG-*aroK* (*p*-CA6). Both strains harbor the expression plasmid pEKEx3-*aroH*_*Ec*_-*tal*_*FjCg*_ for *p*-CA production. The depicted data represent mean values and standard deviation of biological triplicates. **Figure S4.** Effect of genomic integration of codon-optimized gene aroF*_EcCg_ encoding a feedback-inhibition resistant DAHP synthase from *E. coli* on *p*-CA production. **a** Growth (OD_600_), and (**b**) *p*-CA titer of *C. glutamicum* *p*-CA6 (control, circles) and *C. glutamicum* *p*-CA6 IGR9::aroF*_EcCg_ (*p*-CA7) with the integration of aroF*_EcCg_ between cg0432 and cg0435 in a non-coding region under the control of the constitutive *dapA* promotor variant A16 (squares). Both strains harbor the expression plasmid pEKEx3-*aroH*_*Ec*_-*tal*_*FjCg*_ for *p*-CA production. The depicted data represent mean values and standard deviation of biological triplicates. **Figure S5.** Effect of an in-frame deletion of *pyk* encoding pyruvate kinase on *p*-CA production. **a** Growth (OD_600_), and (**b**) *p*-CA titer of *C. glutamicum* *p*-CA7 (control, circles) and *C. glutamicum* *p*-CA7 ∆*pyk* (*p*-CA8) (squares). Both strains harbor the expression plasmid pEKEx3-*aroH*_*Ec*_-*tal*_*FjCg*_ for *p*-CA production. The depicted data represent mean values and standard deviation of biological triplicates. **Figure S6.** Effect of P_i_ limitation on growth and *p*-CA production of *C. glutamicum*. Bacterial growth (OD_600_ [-] (diamonds) and cell dry weight (CDW) [g/L]) (squares)) and *p*-CA titer (circles) is depicted of *C. glutamicum* *p*-CA8 cultivated in defined CGXII medium with varying P_i_ concentrations of (**a**) 13 mM, (**b**) 0.65 mM, (**c**) 0.26 mM, and (**d**) 0.13 mM. The strain harbors the expression plasmid pEKEx3-*aroH*_*Ec*_-*tal*_*FjCg*_ for *p*-CA production. The depicted data represent mean values and standard deviation of biological triplicates. **Figure S7.** Growth of the *C. glutamicum* *p*-CA8 and *C. glutamicum*-RES compared to a co-cultivation of both strains. *C. glutamicum* *p*-CA8 (diamonds), *C. glutamicum*-RES (squares), and both strains in co-cultivation (circles) were cultivated in microtiter plates. The depicted data represent the average and standard deviation of biological triplicates. **Figure S8.** Variation of the inoculation ratios of the *p*-CA- and RES-production strains in co-cultivations. **a **The *p*-CA (**b**) and RES titer of a co-culture of *C. glutamicum* *p*-CA8 and *C. glutamicum* RES was determined in shake flask cultivations. The inoculation ratios of the *p*-CA and RES production strains were adjusted to 1:1 (circles), 0.75:1.25 (squares), 0.5:1.5 (diamonds), and 0.2:1.8 (triangles). The depicted data represent the average and standard deviation of biological triplicates.

## Data Availability

All data generated or analyzed during this study are included in this published article and its supplementary information files.
