# Supplementary material for: Microbial synthesis of the plant natural product precursor p-coumaric acid with Corynebacterium glutamicum
Source: Microb Cell Fact. 2023 Oct 13;22:209. doi: 10.1186/s12934-023-02222-y (PMC10576375; doi:10.1186/s12934-023-02222-y)
Supplement: Supplementary file 1 — Additional file 1: Table S1. Oligonucleotides used in this study. Figure S1. Impact of (ATG→GTG) start codon replacement of pheA and introduction of the point mutation into AroF (AroF-S188C) on growth and metabolite accumulation. Growth (OD600) (diamonds), p-CA titer (circles), and ANT concentration (squares) of (a) C. glutamicum p-CA2, (b) C. glutamicum p-CA2 GTG-pheA, (c) C. glutamicum p-CA2 AroF-S188C and (d) C. glutamicum p-CA2 GTG-pheA AroF-S188C (p-CA3). All strains harbor the expression plasmid pEKEx3-aroHEc-talFjCg for p-CA production. The depicted data represent mean values and standard deviation of biological triplicates. Figure S2. Effect of an in-frame deletion of phdT on p-CA production. (a) Growth (OD600) and (b) p-CA titer of C. glutamicum p-CA4 (control, circles) and C. glutamicum p-CA4 ∆phdT (p-CA5). (squares). Both strains harbor the expression plasmid pEKEx3-aroHEc-talFjCg for p-CA production. The depicted data represent mean values and standard deviation of biological triplicates. Figure S3. Effect of (GTG→ATG) start codon replacement of aroK encoding SAK on p-CA titer and SA accumulation. Growth (OD600, diamonds), p-CA (circles), and SA titer (squares) of (a) C. glutamicum p-CA5 (control) and (b) C. glutamicum p-CA5 ATG-aroK (p-CA6). Both strains harbor the expression plasmid pEKEx3-aroHEc-talFjCg for p-CA production. The depicted data represent mean values and standard deviation of biological triplicates. Figure S4. Effect of genomic integration of codon-optimized gene aroF*EcCg encoding a feedback-inhibition resistant DAHP synthase from E. coli on p-CA production. a Growth (OD600), and (b) p-CA titer of C. glutamicum p-CA6 (control, circles) and C. glutamicum p-CA6 IGR9::aroF*EcCg (p-CA7) with the integration of aroF*EcCg between cg0432 and cg0435 in a non-coding region under the control of the constitutive dapA promotor variant A16 (squares). Both strains harbor the expression plasmid pEKEx3-aroHEc-talFjCg for p-CA production. The depicted da [file 12934_2023_2222_MOESM1_ESM.docx]

**Microbial synthesis of the plant natural product precursor**

***p*-coumaric acid with *Corynebacterium glutamicum***

Mario Mutz^1, 2^, Dominic Kösters^1, 2^, Benedikt Wynands^1^, Nick Wierckx^1^, Jan Marienhagen^1, 2 *^

**^1^** Institute of Bio- and Geosciences, IBG-1: Biotechnology, Forschungszentrum Jülich, D-52425 Jülich, Germany

**^2^** Institute of Biotechnology, RWTH Aachen University, Worringer Weg 3, D-52074 Aachen, Germany

e-mail / ORCID ID:

Mario Mutz: [m.mutz@fz-juelich.de](mailto:m.mutz@fz-juelich.de) 0000-0003-1716-6931

Dominic Kösters: [d.koesters@fz-juelich.de](mailto:d.koesters@fz-juelich.de) 0000-0003-0026-4121

Benedikt Wynands: [b.wynands@fz-juelich.de](mailto:b.wynands@fz-juelich.de) 0000-0001-8599-3205

Nick Wierckx: [n.wierckx@fz-juelich.de](mailto:n.wierckx@fz-juelich.de) 0000-0002-1590-1210

* Corresponding author:

Prof. Dr. Jan Marienhagen, phone: +49 2461 61 2843, e-mail: [j.marienhagen@fz-juelich.de](mailto:j.marienhagen@fz-juelich.de), ORCID ID: 0000-0001-5513-3730

**Tab. S1: Oligonucleotides used in this study**

| oligonucleotide | Sequence [5`🡪3`] |
| --- | --- |
|  |  |
| pK19*mobsacB* check fwd | CAGAGGAAACAGCTATGACCATG |
| pK19*mobsacB* check rev | CGCCAGGCTTTTCCCAGTCACGAC |
| pMKEx2 check fwd | CCCTCAAGACCCGTTTAGAGGC |
| pMKEx2 check rev | TTAATACGACTCACTATAGGGGAATTGTGAGC |
| ∆*phdA* check fwd | GAATCTGAGTGCTCGAGC |
| ∆*phdA* check rev | AATACTCCACACTGTCCCTAGC |
| GTG-*pheA* Up fwd | ATCCCCGGGTACCGAGCTCGTCCGAAGAGTTCGGCTGC |
| GTG-*pheA* Up rev | TGAGCGACGCACCAACTG |
| GTG-*pheA* Down fwd | AACAGTTGGTGCGTCGCTCACGGTTACACAGCTTAACCCGCCGAACTAAGG |
| GTG-*pheA* Down rev | TTGTAAAACGACGGCCAGTGCCGACCAGCCCGCACGCG |
| GTG-*pheA* check fwd | CGGCGACATCATCAACAAGGCG |
| GTG-*pheA* check rev | TGCGTGGTTGAAGTGCGTGC |
| AroF-S188C Up fwd | ATCCCCGGGTACCGAGCTCGATGAGTTCTCCAGTCTCACTC |
| AroF-S188C Up rev | ACATCCCAGAAGCCAGCTG |
| AroF-S188C Down fwd | CCAGCTGGCTTCTGGGATGTGTATGCCAATTGGTTTCAAGAAC |
| AroF-S188C Down rev | TTGTAAAACGACGGCCAGTGTTACTTGGCTGCTGCTCG |
| AroF-S188C check fwd | AACAGAACCGGAGTCGAGCAGC |
| AroF-S188C check rev | TCATACACCGGGTACAGC |
| TrpE-P304S Up fwd | ATGAACTTTTTGGCGCATCCTCTGAGTCCAACCTCAAGTTC |
| TrpE-P304S Up rev | GGATGCGCCAAAAAGTTCATAGG |
| TrpE-P304S Down fwd | ATGAACTTTTTGGCGCATCCTCTGAGTCCAACCTCAAGTTC |
| TrpE-P304S Down rev | TTGTAAAACGACGGCCAGTGCTGGACAAACGCCGAACG |
| TrpE-P304S check fwd | GCCTCAGATGCGGTTGATGAGCG |
| TrpE-P304S check rev | GCAACGTCTCATCGGCTTCAGATTGAGG |
| ∆*phdT* check fwd | GCAACTGTGCCTTTGATTACG |
| ∆*phdT* check rev | CACCGACATCAAACTAGTGGACCCG |
| ATG-*aroK* check fwd | TGAGGCCGGAACCAATGTGGACATC |
| ATG-*aroK* check rev | CGTGAAACCTTGGGCGTGGAAGTGC |
| IGR9::*aroF***_EcCg_* check fwd | TGCTGGCAGAATTCTCCTAATCCGGC |
| IGR9::*aroF***_EcCg_* check rev | AGTGGACAGATATTCTTCGAGATCG |
| ∆*pyk* check fwd | CCATTGGTTCAACGCTAAGG |
| ∆*pyk* check rev | AGGGCATTGATGGAGAAACG |

**Fig. S1: Impact of (ATG🡪GTG) start codon replacement of pheA and** **introduction of the point mutation into AroF (AroF-S188C) on growth and metabolite accumulation.** Growth (OD_600_) (diamonds), p-CA titer (circles), and ANT concentration (squares) of (**a**) C. glutamicum p-CA2, (**b**) C. glutamicum p-CA2 GTG-pheA, (**c**) C. glutamicum p-CA2 AroF-S188C and (**d**) C. glutamicum p-CA2 GTG-pheA AroF-S188C (p-CA3). All strains harbor the expression plasmid pEKEx3-aroH_Ec_-tal_FjCg_ for p-CA production. The depicted data represent mean values and standard deviation of biological triplicates.

**Fig. S2: Effect of an in-frame deletion of phdT on p-CA production.** (**a**) Growth (OD_600_) and (**b**) p-CA titer of C. glutamicum p-CA4 (control, circles) and C. glutamicum p-CA4 ∆phdT (p-CA5). (squares). Both strains harbor the expression plasmid pEKEx3-aroH_Ec_-tal_FjCg_ for p-CA production. The depicted data represent mean values and standard deviation of biological triplicates.

**Fig. S3: Effect of (GTG🡪ATG) start codon replacement of aroK encoding SAK on p-CA titer and SA accumulation.** Growth (OD_600_, diamonds), p-CA (circles), and SA titer (squares) of (**a**) C. glutamicum p-CA5 (control) and (**b**) C. glutamicum p-CA5 ATG-aroK (p-CA6). Both strains harbor the expression plasmid pEKEx3-aroH_Ec_-tal_FjCg_ for p-CA production. The depicted data represent mean values and standard deviation of biological triplicates.

**Fig. S4: Effect of genomic integration of codon-optimized gene aroF*_EcCg_ encoding a feedback-inhibition resistant DAHP synthase from E. coli on p-CA production. (a)** Growth (OD_600_), and (**b**) p-CA titer of C. glutamicum p-CA6 (control, circles) and C. glutamicum p-CA6 IGR9::aroF*_EcCg_ (p-CA7) with the integration of aroF*_EcCg_ between cg0432 and cg0435 in a non-coding region under the control of the constitutive dapA promotor variant A16 (squares). Both strains harbor the expression plasmid pEKEx3-aroH_Ec_-tal_FjCg_ for p-CA production. The depicted data represent mean values and standard deviation of biological triplicates.

**Fig. S5: Effect of an in-frame deletion of pyk encoding pyruvate kinase on p-CA production. (a)** Growth (OD_600_), and (**b**) p-CA titer of C. glutamicum p-CA7 (control, circles) and C. glutamicum p-CA7 ∆pyk (p-CA8) (squares). Both strains harbor the expression plasmid pEKEx3-aroH_Ec_-tal_FjCg_ for p-CA production. The depicted data represent mean values and standard deviation of biological triplicates.

**Fig. S6: Effect of P_i_ limitation on growth and p-CA production of C. glutamicum.** Bacterial growth (OD_600_ [-] (diamonds) and cell dry weight (CDW) [g/L]) (squares)) and p-CA titer (circles) is depicted of C. glutamicum p-CA8 cultivated in defined CGXII medium with varying P_i_ concentrations of (**a**) 13 mM, (**b**) 0.65 mM, (**c**) 0.26 mM, and (**d**) 0.13 mM. The strain harbors the expression plasmid pEKEx3-aroH_Ec_-tal_FjCg_ for p-CA production. The depicted data represent mean values and standard deviation of biological triplicates.

**Fig. S7: Growth of the C. glutamicum p-CA8 and C. glutamicum-RES compared to a co-cultivation of both strains.** C. glutamicum p-CA8 (diamonds), C. glutamicum-RES (squares), and both strains in co-cultivation (circles) were cultivated in microtiter plates. The depicted data represent the average and standard deviation of biological triplicates.

**Fig. S8: Variation of the inoculation ratios of the p-CA- and RES-production strains in co-cultivations.** (**a**) The p-CA (**b**) and RES titer of a co-culture of C. glutamicum p-CA8 and C. glutamicum RES was determined in shake flask cultivations. The inoculation ratios of the p-CA and RES production strains were adjusted to 1:1 (circles), 0.75:1.25 (squares), 0.5:1.5 (diamonds), and 0.2:1.8 (triangles). The depicted data represent the average and standard deviation of biological triplicates.
